# The gut microbiota and endometriosis: From pathogenesis to diagnosis and treatment

**DOI:** 10.3389/fcimb.2022.1069557

**Published:** 2022-11-24

**Authors:** Rui Qin, Gengren Tian, Junbao Liu, Lu Cao

**Affiliations:** ^1^ Department of Gynecology, China-Japan Union Hospital of Jilin University, Changchun, Jilin, China; ^2^ Department of Neurosurgery, China-Japan Union Hospital of Jilin University, Changchun, Jilin, China; ^3^ Department of Obstetrics, China-Japan Union Hospital of Jilin University, Changchun, Jilin, China

**Keywords:** endometriosis, gut microbiota, inflammation, estrogen, probiotics

## Abstract

Endometriosis is a common gynecological disease, that often leads to pain and infertility. At present, the specific pathogenesis of endometriosis has not been clarified, but it may be closely related to an imbalance of sex hormones in the body, ectopic hyperplasia stimulated by immune inflammation, and invasion and escape based on tumor characteristics. Gut microbiota is associated with many inflammatory diseases. With the further study of the gut microbiota, people are paying increasing attention to its relationship with endometriosis. Studies have shown that there is an association between the gut microbiota and endometriosis. The specific ways and mechanisms by which the gut microbiota participates in endometriosis may involve estrogen, immune inflammation, and tumor characteristics, among others. Therefore, in the future, regulating gut microbiota disorders in various ways can help in the treatment of endometriosis patients. This study reviewed the research on the gut microbiota and endometriosis in order to provide ideas for clinical diagnosis and treatment.

## Introduction

Endometriosis is an estrogen dependent chronic inflammatory disease, that often leads to pain and infertility (Ozkan et al., 2008). The prevalence of endometriosis in women of childbearing age is 10%~15%. For women with abdominal pain and/or unexplained infertility, the incidence is as high as 40%~60% (Ilangavan and Kalu, 2010). To data, there are many theories about the pathogenesis of endometriosis, including menstruation countercurrent. However, none of them fully elucidate the specific mechanism, which has become the major reason limiting breakthrough progress in endometriosis treatment. In recent years, with the development of genomics research and high-throughput sequencing technology, a large number of studies have shown that the human microbiota has a significant relationship with female reproductive health (Martin, 2012; Nelson et al., 2016). Studies have showed that the gut microbiota is associated with many inflammatory diseases and that there is an association between the gut microbiota and endometriosis (Zhu et al., 2020; Talwar et al., 2022). The mechanism by which the the gut microbiota affects endometriosis may involve estrogen, immunity, inflammation, and tumor characteristics, etc (Salliss et al., 2021). Therefore, targeting the gut microbiota may help in the treatment of endometriosis in the future.

## Dysbiosis of the gut microbiota in patients with endometriosis

The gut microbiota not only plays an important role in food metabolism and intestinal physiology, and homeostasis imbalance in the gut microbiota can lead to the occurrence and development of various diseases (Xu et al., 2020). Studies have found that gut microbiota disorders can not only cause inflammatory bowel disease, irritable bowel syndrome, and colon cancer in the intestinal system, but can also induce diseases in the extraintestinal system, such as diabetes, mastitis, and polycystic ovary syndrome (Baothman et al., 2016; Sun and Kato, 2016; Zhao C. et al., 2021). Endometriosis is considered to be closely related to immune disorders because its characteristics are similar to those of autoimmune diseases such as decreased apoptosis, increased cytokine levels and abnormal cell-mediated pathways (Aznaurova et al., 2014; Ahn et al., 2015). Meanwhile, the interaction between the immune system and gut microbiota plays a fundamental role in maintaining immune homeostasis (Wu and Wu, 2012). Therefore, many scholars have conducted in-depth research on the relationship between endometriosis and the gut microbiota.

Yu et al. investigated the gut microbiota profile associated with patients with stage 3/4 endometriosis. They found that the diversity of the gut microbiota in patients with endometriosis decreased and the ratio of Firmicutes/Bacteroidetes increased. Prevotella_7 was the most abundant taxon in patients with endometriosis and Coprococcus_2 was the most abundant taxon in controlled women. Meanwhile, the abundances of Actinobacteria, Cyanobacteria, Saccharibacteria, Fusobacteria, and Acidobacteria were significantly increased in patients with endometriosis. The LEfSe analysis demonstrated that Blautia, Bifdobacterium, Dorea, and Streptococcus abundances are related to the inflammatory and serum hormone levels (Shan et al., 2021). Bulent et al. found that gut microbiota composition was changed in patients with endometriosis. Firmicutes was the major phylum and Bacteroidetes was the second most abundant phylum in the gut microbiota. Firmicutes was increased in patients with endometriosis. Furthermore, they found that *Actinobacteria*, *Bacteroidetes*, *Firmicutes*, *Fusobacteria*, *Proteobacteria*, *and Verrucomicrobia* in the gut were correlated with concentrations of urinary estrogens (Ata et al., 2019). A recent study by Bodil et al. compared the gut microbiota between patients with endometriosis and healthy controlled women. They found that both the alpha and beta diversities were changed. And Bacilli, Bacteroidia, Clostridia, Coriobacteriia, and Gammaproteobacter levels differed between the endometriosis group and control group. In experimental animals, it was found that there was a significant difference in β diversity. Firmicutes was enriched in the model group, while Bacteroides was enriched in the control group (Hantschel et al., 2019). Cao et al. also found that the Firmicutes/Bacteroides ratio in the body increased after successful modeling of endometriosis in rats, indicating that endometriosis causes a gut microbiota imbalance (Cao et al., 2020).

## Gut microbiota biomarkers for diagnosing endometriosis

Because the onset of endometriosis is hidden and a diagnosis can only be made through invasive procedures, such as laparoscopy and histopathology, the current clinical diagnosis approach lacks sensitive and specific biological indicators, leading to a delay in diagnosis and treatment of the disease, which seriously affects the quality of life of women (Agarwal et al., 2019). Therefore, the search for noninvasive biomarkers is of great significance for the clinical diagnosis and treatment of endometriosis. Some diagnostic markers in the serum, urine, menstrual blood and other body fluids of women with endometriosis have been reported (Anastasiu et al., 2020). The use of these markers has the advantages of a simple, rapid and noninvasive diagnosis, and thus has become an important direction for diagnosis of the disease. Associations between gastrointestinal and genital tract microbial health and endometriosis have been identified. Small molecular metabolites derived from gut microbiota have the potential for diagnosing endometriosis. Second bile acid biosynthesis and alpha-linolenic acid (ALA) metabolism were the significant enrichment pathways of gut microbiota in patients of endometriosis. A recent study demonstrated that the predictive value of vaginal microbiota in endometriosis may not be as important as that of gut microbiota, which to some extent brings a new direction in the study of endometriosis. *Ruminococcus* and *Pseudomonas* have been identified as potential biomarkers in intestinal and peritoneal fluids for diagnosing endometriosis (Huang L. et al., 2021).

## Role of the gut microbiota in the pathogenesis of endometriosis

### Gut microbiota and estrogen

Endometriosis is an estrogen-dependent disease, that is characterized by the presence of endometrial glands and stromal cells outside the uterus (Ferrero et al., 2014). Estrogen plays an important role in maintaining female reproductive system development (Jeon et al., 2016). Estrogen can regulate the microenvironment of the female lower genital tract by increasing epithelial thickness, glycogen levels and mucus secretion, and indirectly reducing vaginal pH by increasing Lactobacillus abundance and lactic acid levels (Baker et al., 2017). Studies have found that estrogen can induce proliferative diseases such as endometriosis, endometrial cancer, and hysteromyoma by stimulating the proliferation of female genital epithelial cells (Buchanan et al., 1998; Merrheim et al., 2020). The metabolism of estrogen mainly occurs in the liver. The liver can produce sex hormone binding globulin, and the combination of sex hormone binding globulin and estrogen can lead to loss of estrogen biological activity (Phelps et al., 2019). The gut microbiota can secrete β-glucuronidase and β-glucosidase, and these products can promote the degradation of estrogen, thus increasing the reabsorption of free estrogen, and improving the level of estrogen in the circulation (Possemiers et al., 2011; Kwa et al., 2016). Analysis of the microbial genome shows that multiple bacterial genera in the gut microbiota can produce β-glucuronidase, including *Bacteroid, Bifidobacterium, Escherichia coli* and *Lactobacillus* (Beaud et al., 2005). Notably, in the feces of endometriosis patients, the *Escherichia coli* content is significantly increased (Leonardi et al., 2020). The above studies confirmed that the gut microbiota can lead to an increase in circulating estrogen levels, which is helpful in creating a high-estrogen environment for endometriosis progression (Qi et al., 2021). However, the factors that stimulate the production of β-glucuronidase by specific gut microbiota in the pathogenesis of endometriosis, the relationship between the gut microbiota and female upper reproductive tract microbiota, and whether they play a synergistic role in the pathogenesis of endometriosis still need further research.

### Gut microbiota and inflammation

Although endometriosis is an estrogen dependent disease, some studies have found that the growth of ectopic lesions continues even in ovariectomized animals, indicating that in addition to ovarian steroids, the innate immune system in the pelvic environment can also regulate the growth of ectopic lesions in endometriosis (Khan et al., 2009). The natural immune cells of mammals (such as macrophages and dendritic cells) can be activated by microbial components (non self), such as endotoxin or lipopolysaccharide from gram-negative bacteria (Ramirez-Pavez et al., 2021). Research shows that the inflammatory microenvironment is closely related to the occurrence of endometriosis (Bruner-Tran et al., 2013). The inflammatory response is the central process in the development of endometriosis, leading to pain, tissue remodeling, fibrosis, adhesion formation and infertility (Mu et al., 2018). A series of changes in inflammatory factors, cytokines and chemokines are observed in the focus and peritoneal fluid of patients with endometriosis, including both proinflammatory and anti-inflammatory components (Machairiotis et al., 2021). The levels of the proinflammatory factors IL-1β, IL-18, and TGF-β are increased in the peritoneal fluid. When the inflammatory activity decreases, the level of the anti-inflammatory factor IL-37 increases (Zhou et al., 2019). When the body is unable to remove inflammatory substances, an inflammatory state is induced, which may spread from the region of inflammation to the whole body and have a long-term impact on the immune system (Symons et al., 2018). One important factor in the pathogenesis of endometriosis is that deficiency of the immune system leads to difficulty in clearing ectopic endometrial tissue. The increase in the levels of proinflammatory factors, anti-inflammatory factors and immune cells reflects an imbalance in the regulation of inflammation and anti-inflammatory processes, as well as changes in intestinal microbiota, intestinal permeability and other immune regulatory processes (Laschke and Menger, 2016).

Recently, studies have demonstrated that the gut microbiota may interact with cytokines abnormally expressed in endometriosis lesions, peritoneal fluid and peripheral blood (Yuan et al., 2018). Increased *Escherichia coli* and *Shigella* in the gut were observed in patients with endometriosis (Quaranta et al., 2019). Additionally, the level of LPS in the gut and serum is also increased in patients with endometriosis (Ni et al., 2020). Studies have shown that both initial inflammatory mediators (lipopolysaccharide) and secondary inflammatory mediators (cytokines/growth factors) participate in the development of endometriosis, and that TLR are expressed in macrophages and other dendritic cells (Azuma et al., 2017). Other studies have shown that macrophages can promote the release of inflammatory mediators under lipopolysaccharide stimulation, thereby promoting the inflammatory infiltration, proliferation and angiogenesis observed in endometriosis (Rana et al., 1996; Jeljeli et al., 2020).

### Targeting the gut microbiota for the treatment of endometriosis

Endometriosis is a chronic disease with a recurrence rate of 10%-15% one year after conservative surgery. At the 5-year follow-up, the recurrence rate was 40%-50% (Berlanda et al., 2010). Women with endometriosis will experience severe pain during menstruation and sexual intercourse, which can not only cause health problems, but also interfere with normal activities during work and leisure time (Huntington and Gilmour, 2005). In addition to pain and infertility, endometriosis has a huge negative impact on women’s social functions, work and employment (Facchin et al., 2015). Treatment of endometriosis involves conservative or radical surgery, or medical therapies. Therefore, finding a safe and effective way to prevent and treat endometriosis has become a global problem, especially for women of reproductive age with endometriosis. Gut microbiota imbalance has many adverse effects on the body, and correcting the gut microbiota imbalance to restore its normal functional state provides another option for the treatment of many diseases, including endometriosis. Regulation of gut microbiota by means of antibiotics, fecal bacteria transplantation, probiotics, nutrients, etc., may provide new ideas for the clinical treatment of endometriosis (Xu et al., 2015).

### Metabolites of gut microbiota

Gut microbiota imbalance has been found to be closely related to a reduction in short-chain fatty acid production (Macfabe, 2012; Melbye et al., 2019). Butyric acid is a short-chain fatty acid that plays an important role in maintaining the intestinal barrier, inhibiting immune function and optimizing mitochondrial function (Zhao Z. H. et al., 2021; Rekha et al., 2022). In addition, butyrate can also inhibit the immune response caused by intestinal biological imbalance (Ma et al., 2020). Some scholars have found that the gut microbiota can cause depression by reducing the short-chain fatty acid butyrate; patients with endometriosis have a relatively high risk of depression, and women with chronic pelvic pain usually have a higher degree of depression than those without pain (Silva et al., 2020). Furthermore, a recent study showed that gut microbiota-derived butyrate can protect mice against endometriosis by regulating G-protein-coupled receptors (Chadchan et al., 2021).

A study on the correlation between fecal metabonomics and gut microbiota in mice with endometriosis found that chenodeoxycholic acid (CDCA) and ursodeoxyl content increased and the abundance of linolenic acid (ALA) decreased (Ni et al., 2020). Meanwhile, another study demonstrated that ALA reduced the inflammatory response by inhibiting the accumulation of nitrite and prostaglandin E2 (PGE2) (Ni et al., 2021). In addition, ALA can inhibit the inflammatory response of M1 macrophages (Pauls et al., 2018). Moreover, ALA can improve the abdominal inflammatory environment and reduce the level of LPS in mice with endometriosis (Ni et al., 2021). The above two studies suggest that metabolic changes may play an important role in the pathogenesis of endometriosis and that some metabolites of gut microbiota may have the potential to treat endometriosis.

### Probiotics and prebiotics

Gut microbiota can be divided into symbiotic bacteria, probiotics and pathogenic bacteria. Gut microbiota disorder is considered to be a decrease in the number of probiotics in the body (Barengolts, 2016; Feng et al., 2018). Therefore, many scholars have carried out studies on the addition of probiotics. Karamali et al. conducted a randomized, double-blind controlled trial in women with polycystic ovary syndrome (Karamali and Gholizadeh, 2022). They found that probiotic supplementation in women with polycystic ovary syndrome could significantly improve serum sex hormone binding globulin and plasma total antioxidant capacity, significantly reduce total testosterone level, and benefit the body (Karamali and Gholizadeh, 2022). Vitellio et al. found that adding *Bifidobacterium longum* alleviated the symptoms of lactose intolerance patients (Vitellio et al., 2019). Furthermore, probiotics have also been used for the treatment of endometriosis (Chenoll et al., 2019). *Lactobacillus gasseri* has protective effects against endometriosis by inhibiting inflammation and the development of ectopic endometrial cells (Itoh et al., 2011; Uchida and Kobayashi, 2013).

In recent years, increasing attention has been given to botanical drugs, which include not only traditional Chinese medicine, but also various plant extracts (Li, 2002; Liu and Wang, 2008). Botanical drugs have been widely studied for their role in the treatment of endometriosis, and are considered as a good strategy with few side effects and the benefit of fertility retention (Flower et al., 2011; Bina et al., 2019). At present, the most commonly studied botanical drug types include plant extracts, plant-derived bioactive compounds and Chinese herbal medicine (Farnsworth, 1990). Plant extracts, including pueraria lobata and black garlic extracts, have been widely confirmed to play an anti-inflammatory, antiangiogenic and antioxidant roles in endometrial cells. Bioactive substances, including resveratrol, catechin, and curcumin, are believed to modulate a variety of signaling pathways and have therapeutic effects in endometriosis (Kamal et al., 2021; Hipolito-Reis et al., 2022; Madanes et al., 2022). Although botanical drugs have shown good “potential” *in vitro* and in animal experiments, the data from clinical double-blind randomized controlled trials are still insufficient. The clinical effects and safety of these drugs need to be further evaluated and verified.

### Dietary regulation

Different dietary components deliver different carbohydrates and phytonutrients to the colon, causing different microbial changes (Yalcin Bahat et al., 2022). Wheat bran free foods, such as fruits, vegetables, meat, eggs, and milk can change the distribution of the gut microbiota and regulate intestinal permeability (Lazar et al., 2019; Campmans-Kuijpers and Dijkstra, 2021). The addition of probiotics can inhibit the immune inflammatory cascade reaction in patients with depression and improve their mental state. A large body of studies have demonstrated that dietary factors are associated with endometriosis risk (Parazzini et al., 2013; Jurkiewicz-Przondziono et al., 2017). Trans-unsaturated fatty acids and red meat can increase the risk of endometriosis (Missmer et al., 2010). Women who consume a large amount of fruits, vegetables, dairy products, and omega-3 fatty acids have an attenuated risk of endometriosis (Samaneh et al., 2019). The intake of antioxidants and a combination of vitamins and minerals may have protective effects against endometriosis (Guney et al., 2007). Recent studies have demonstrated that ω-3 polyunsaturated fatty acids can change the composition of gut microbiota (Watson et al., 2018). Adding ω-3 polyunsaturated fatty acids and probiotics can effectively prevent osteoporosis, obesity and diabetes (Bi et al., 2017; Wang et al., 2022). ω-3 polyunsaturated fatty acids were found to attenuate the inflammation in a mouse model of endometriosis (Pereira et al., 2019). In addition, women who consume a large amount of ω-3 polyunsaturated fatty acids have a lower risk of developing endometriosis (Brasky et al., 2015). It is speculated that regulation of the gut microbiota through diet may help prevent endometriosis.

### Fecal microbiota transplantation

Fecal microbiota transplantation (FMT) refers to a standardized treatment in which the feces of healthy people, and therefore the acquired functional flora, is transplanted into the gastrointestinal tract of patients with dysbacteriosis to rebuild the gut microbiota of patients and treat disease (Kelly et al., 2015; Nigam et al., 2022). At present, FMT has been applied for the treatment of gastrointestinal diseases, blood diseases, neuropsychiatric diseases, chronic hepatitis B, metabolic syndrome, drug-resistant bacterial infections and other diseases (Brandt and Aroniadis, 2013; Kim and Gluck, 2019). In 2013, FMT was written into the clinical guidelines of the United States for the treatment of recurrent *Clostridium difficile* infection (Cammarota et al., 2014). The clinical efficacy of FMT has been verified in many diseases (Bowman et al., 2015). Recently, studies have shown that FMT can be used for the treatment of female reproductive tract diseases (Quaranta et al., 2019; Huang J. et al., 2021). Due to the role of gut microbiota in the development of endometriosis, FMT could be an innovative treatment option for the treatment of endometriosis.

## Conclusions

Considering the different phenotypes of endometriosis and the diverse clinical manifestations, in the future, we can further study the different characteristics of microbiota in different endometriosis patients and the role of their metabolites in the pathogenesis of endometriosis. We hope to clarify the relationship between the gut microbiota and endometriosis by analyzing the characteristics and metabolites of the gut microbiota in patients with endometriosis and provide new ideas for the prevention, diagnosis and treatment of endometriosis. In the future, we should further study the correlation between the gut microbiota and endometriosis from the aspects of systemic immunity, metabolism, and tumors, among other perspectives. At the same time, the gut microbiota can be adjusted by means of antibiotics, fecal microbial transfer, probiotics, nutrients and other means to intervene in endometriosis, providing new ideas for the clinical treatment of endometriosis.

## Author contributions

LC and RQ wrote the manuscript; JL and GT revised the review. All authors contributed to the article and approved the submitted version.

## Conflict of interest

The authors declare that the research was conducted in the absence of any commercial or financial relationships that could be construed as a potential conflict of interest.

## Publisher’s note

All claims expressed in this article are solely those of the authors and do not necessarily represent those of their affiliated organizations, or those of the publisher, the editors and the reviewers. Any product that may be evaluated in this article, or claim that may be made by its manufacturer, is not guaranteed or endorsed by the publisher.
